# Childbirths and the Prevalence of Potential Risk Factors for Adverse Perinatal Outcomes among Asylum Seekers in The Netherlands: A Five-Year Cross-Sectional Study

**DOI:** 10.3390/ijerph182412933

**Published:** 2021-12-08

**Authors:** Julia B. Tankink, Anouk E. H. Verschuuren, Ineke R. Postma, Peggy J. A. van der Lans, Johanna P. de Graaf, Jelle Stekelenburg, Annelies W. Mesman

**Affiliations:** 1 Department of Obstetrics and Gynecology, Erasmus University Medical Center, 3000 CA Rotterdam, The Netherlands; j.degraaf@erasmusmc.nl; 2 Global Health Unit, Department of Health Sciences, University Medical Center Groningen, University of Groningen, Hanzeplein 1, 9713 GZ Groningen, The Netherlands; j.stekelenburg@umcg.nl; 3 Department of Obstetrics and Gynecology, University Medical Center Groningen, University of Groningen, Hanzeplein 1, 9713 GZ Groningen, The Netherlands; i.r.postma@umcg.nl; 4 Department of Obstetrics and Gynecology, Hospital Twente ZGT/MST, 7512 KZ Enschede, The Netherlands; p.vanderlans@mst.nl; 5 Department of Obstetrics and Gynecology, Medical Center Leeuwarden, Henri Dunantweg 2, 8934 AD Leeuwarden, The Netherlands; 6 Netherlands Association for Community Health Services (GGD GHOR Nederland), Zwarte Woud 2, 3524 SJ Utrecht, The Netherlands; amesman@ggdghor.nl

**Keywords:** asylum seekers, pregnancy, risk factors, refugees, perinatal health, health inequities, teenage birthrate, length of stay, relocations

## Abstract

This five-year cross-sectional study mapped the prevalence of several known risk factors for adverse perinatal outcomes in asylum-seeking women in The Netherlands. Characteristics of 2831 registered childbirths among residents of asylum seekers centers (ASCs) in The Netherlands from 2016 to 2020 were included. Results showed a high general and teenage birthrate (2.15 and 6.77 times higher compared to the Dutch, respectively). Most mothers were pregnant upon arrival, and the number of births was highest in the second month of stay in ASCs. Another peak in births between 9 and 12 months after arrival suggested that many women became pregnant shortly after arrival in The Netherlands. Furthermore, 69.5 percent of all asylum-seeking women were relocated between ASCs at least once during pregnancy, which compromises continuity of care. The high prevalence of these risk factors in our study population might explain the increased rate of adverse pregnancy outcomes in asylum seekers compared to native women found in earlier studies. Incorporating migration-related indicators in perinatal health registration is key to support future interventions, policies, and research. Ultimately, our findings call for tailored and timely reproductive and perinatal healthcare for refugee women who simultaneously face the challenges of resettlement and pregnancy.

## 1. Introduction

Health equity among women and their babies during pregnancy, childbirth and the postnatal period is under serious pressure in a migration context [1,2]. Asylum seekers represent a specific migrant population who may face higher rates of several adverse maternal health outcomes, such as postnatal complications and postpartum depression, as well as adverse perinatal outcomes, such as stillbirth and low birthweight, compared to native populations [3,4,5,6].

In The Netherlands, a recent study demonstrated a 7 times higher risk of perinatal mortality (defined as death between 22 weeks of pregnancy and 7 days postpartum) among asylum seekers as compared to Dutch women in a regional hospital [6]. Another Dutch study reported a maternal mortality ratio (MMR) of 69.33 per 100,000 births among asylum seekers, which was 10.08 times that of the native population (95% CI 8.02 to 12.83) [7]. In addition, asylum seekers had a 4.5 times greater risk of severe acute maternal morbidity (RR 4.5; 95% CI 3.3–6.1) compared to the general population. This risk remained 3.6 times higher when comparing asylum seekers to other non-Western immigrant groups (RR 3.6; 95% CI 2.6–5.0) [8].

A complex interplay of social, medical, and migration-related determinants places asylum seekers in a particularly vulnerable situation as expectant mothers [3,4,5,9]. In the process of forced migration, women may be exposed to gender-based violence, other types of potential trauma and perilous living conditions in refugee camps or on the streets [10,11]. Healthcare, including antenatal care, does not always come timely, and continuity of care is often compromised while women relocate to or within the country of resettlement [9,12]. Once seeking or receiving care, cultural differences and language barriers can hamper effective communication and understanding between care providers and pregnant women [13,14].

Previous research identified risk factors for severe acute maternal morbidity in asylum seekers, including single motherhood, low socio-economic status, short duration of stay in The Netherlands and a major language barrier [8]. In addition, pregnancies may be complicated by preexistent disease, such as HIV infection or perinatal mental health disorders [15,16,17]. The stress associated with an uncertain residence status, lengthy asylum procedures or financial hardship may further explain why asylum seekers are disadvantaged in perinatal health [12].

Given the strong indication of health disparities between asylum-seeking and native women, there is ample reason to monitor asylum seekers’ perinatal health status and pregnancy outcomes. However, asylum seekers remain a relatively understudied population, as hospital records and national perinatal registries in most countries lack migration-related indicators [18,19]. Therefore, the possibilities to identify and study different migrant populations are limited. In The Netherlands, asylum seekers with a length of stay shorter than six months will generally not have a social security number and therefore cannot easily be traced in national perinatal registry data.

To develop focused interventions and target perinatal health inequities, more insight into the population and reproductive health needs of residents in asylum seekers centers (ASCs) is key. With the use of a unique database, this study aimed to present an overview of childbirths among women in Dutch ASCs and assess the prevalence of several previously described risk factors for adverse perinatal outcomes.

## 2. Materials and Methods

This was a five-year cross-sectional study which used data from the Dutch Central Agency for the Reception of Asylum Seekers (in Dutch “Centraal Orgaan opvang Asielzoekers; COA”).

### 2.1. Setting

In The Netherlands, the COA is the governmental organization which is nationally responsible for the accommodation and assistance of asylum seekers. The COA provides asylum seekers with housing while the immigration services process their asylum request. COA locations include 2 central reception centers and around 60 asylum seeking centers (ASC). After first registration and short-term stay in a central reception center, asylum seekers will be relocated to an ASC. Subsequent relocations between ASCs may occur in the context of the asylum procedure or for a variety of other reasons, such as limited capacity or closure of centers, family reunification or special care needs. At present, the Dutch guideline of perinatal healthcare for asylum seekers advises against relocation of pregnant asylum seekers between 34 weeks of gestation and 6 weeks postpartum [20]. Hereafter, all COA locations will be referred to as ASCs.

### 2.2. Healthcare and Perinatal Care for Asylum Seekers

**Figure 1:**
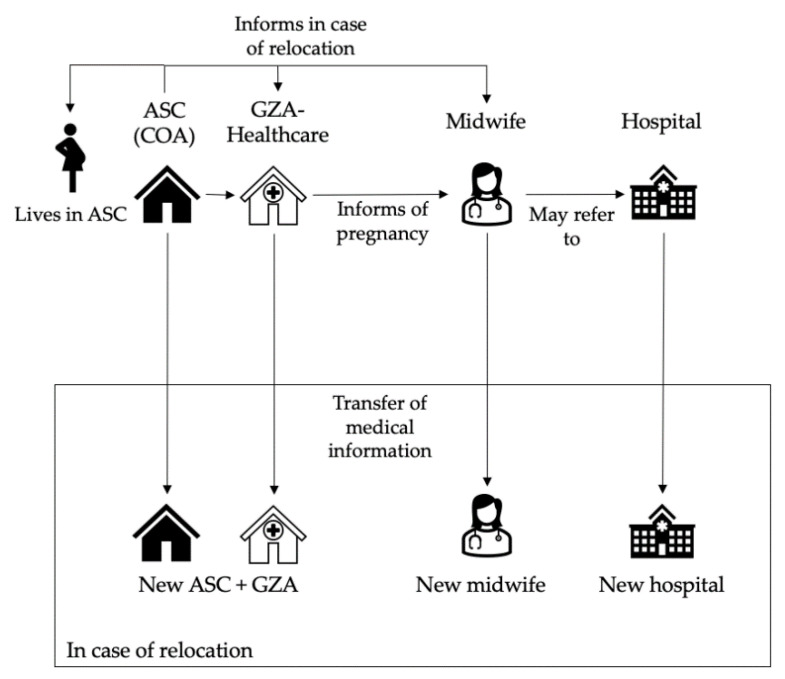
Organization of antenatal care and relocations of pregnant asylum seekers in The Netherlands. ASC = asylum seekers center; COA = Central Agency for the reception of Asylum seekers; GZA = GZA Healthcare (health care center of contracted primary care provider for asylum seekers).

In The Netherlands, healthcare is covered by governmental insurance for asylum seekers. Asylum seekers receive primary healthcare from a contracted organization (GZA Healthcare) which has health centers in most ASCs, while perinatal care is provided by midwifery practices located near ASCs. In the Dutch system, all pregnant women, including asylum seekers, receive midwife-led care unless they are referred to gynecologists/obstetricians in case of (threatening) complications or “high-risk” pregnancy. In case of the relocation of asylum seekers during pregnancy, all medical care and patient history is transferred to new care providers at the next GZA, midwifery practice and/or hospital (see Figure 1). The specific pathways and responsibilities in birth care for asylum seekers have been documented in a national guideline for all stakeholders involved [20].

### 2.3. Study Population

Our study population included all women accommodated in an ASC at the time of childbirth between 1 January 2016 and 1 January 2021. Mothers were included regardless of the status of their request for asylum (in process, approved or denied). As undocumented women are legally entitled to housing in ASCs from six weeks before the due date to at least six weeks after childbirth, these women were also included in the sample. In this study we will further refer to our study population as asylum seekers.

### 2.4. Data Collection

The administrative system of the COA contained demographic information, housing details and information about childbirths of women in ASCs. Childbirths included all babies born alive after 22 weeks of gestation. Multiple pregnancies were considered as one birth in this study; if one mother gave birth more than once during the study period, births and maternal characteristics were included separately. The COA anonymized data and provided the following data for each birth: maternal age in years (calculated at the time of birth); date of registration at an ASC; country of origin; number of relocations between COA locations within nine months prior to birth and registration with a husband or partner (yes/no). Lack of partner registration in an ASC did not necessarily mean a partner was not involved, for example, because partners could have stayed behind in the country of origin. In order to calculate birthrates, the COA provided the total number of asylum seekers in ASCs by sex, age, and country of origin on every first day of the month during the study period.

Data on the Dutch population were derived from Statistics Netherlands (in Dutch: Centraal Bureau voor de Statistiek, CBS) [21].

### 2.5. Data Processing

From the COA dataset, we derived our main study outcomes, including birthrate, teenage birthrate, number of relocations during pregnancy, length of stay and registered with partner (yes/no). We calculated birthrates per 1000 person years in female asylum seekers of fertile age, as previously described by Goosen et al. [22]. These person years were estimated through the total number of female asylum seekers aged 15–49 accommodated in ASCs each month. Birthrates were compared to Dutch population birthrates, which were defined as the number of livebirths per 1000 women aged 15–49.

Using the date of registration at an ASC, we calculated length of stay at the time of birth. Categories of 0–9, 9–12 and 12+ months of stay at childbirth were chosen to estimate the number of women who were pregnant upon arrival in The Netherlands. Teenage births were defined as births among mothers aged below 20 years on the day of birth. We grouped countries of origin in accordance with UNHCR worldwide operations [23].

### 2.6. Statistical Analysis

Descriptive statistics were applied to report all outcomes. As our study included the entire population of women who gave birth while registered in an ASC in The Netherlands during the study period (instead of a sample), inferential statistics were not considered appropriate.

### 2.7. Ethical Considerations

This study was approved by an acknowledged medical ethical committee (MEC-2021-0552, Erasmus MC Rotterdam) and was not subject to the Medical Research Involving Human Subjects Act in The Netherlands. Regarding privacy issues, all data were retrieved and handled anonymously.

## 3. Results

The total number of registered newborns in the study period was 2933. Of all births, we excluded 11 because the registration date of the mother was after the date of birth. Thus, 2922 births were included in the birthrate calculations. From 2016 to 2020, 170 mothers gave birth to 2 children and 4 mothers gave birth to 3 children. After deduplication of 41 twin births, a total of 2881 births remained. Maternal characteristics were considered for 2831 childbirths (among 2694 unique mothers), as an additional 50 births were excluded due to missing information of the mother.

### 3.1. Childbirths and Maternal Characteristics

The number of births varied between years, with 778 births in 2016, 452 in 2017, 427 in 2018, 652 in 2019 and 572 in 2020 (see Appendix A). Of all 2831 births for which maternal characteristics were available, 319 births (11.3 percent) were registered among undocumented women residing in an ASC at the time of childbirth. The age of women ranged from 15 to 51 years old at the time of birth, and most women originated from different African regions (33.8 percent from Middle East/North Africa, 18.7 percent from East/Horn of Africa and 16.2 percent from West/Central Africa) (see Table 1). The most common countries of origin included Syria, Nigeria, Eritrea, Iraq, Iran, and Afghanistan (see Appendix B).

### 3.2. Length of Stay and Number of Relocations

**Figure 2:**
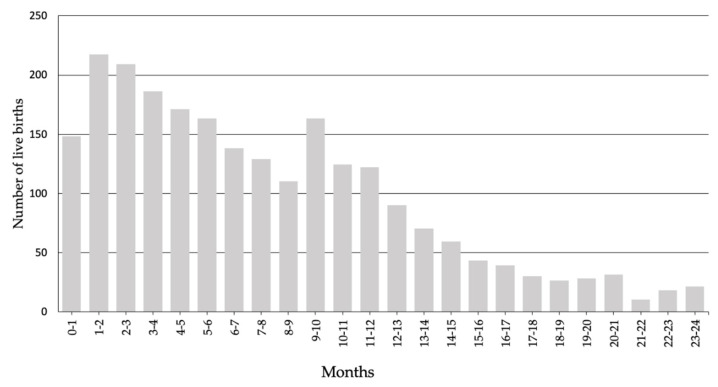
Distribution of live births by the mothers’ length of stay in an ASC before giving birth (from 0–24 months).

Most asylum-seeking women (52.0 percent) gave birth within 9 months after arrival in an ASC; 14.4 percent of women gave birth between 9 and 12 months after arrival and 33.6 percent stayed in ASCs for more than 12 months before giving birth (see Table 1). Overall, the largest number of women gave birth in the second month after arrival. From the second month onwards, the number of births showed a downward trend up until 24 months after arrival. Between 9 and 12 months, there was a deviation from the trend due to a peak in births (see Figure 2).

The number of relocations during pregnancy varied between 0 and 7 times. Of all asylum-seeking women, 69.5 percent were relocated once or more, and 28.2 percent were relocated two times or more during pregnancy (See Table 1). Of all relocations, 40.1 percent took place between a central reception center and an ASC. Of the women who were relocated more than 3 times during pregnancy, 104 women were relocated 4 times, 20 women 5 times, 3 women 6 times and 1 woman 7 times.

### 3.3. Birthrates and Region of Origin

The average birthrate in the asylum-seeking population was 96.77 births per 1000 women of fertile age. This rate was 2.15 times higher compared to the Dutch population, which had 44.99 live births per 1000 women of fertile age (95% CI 44.90–45.09) [22]. Birthrates varied between different regions of origin. Women from West/Central Africa and Southern Africa had a relatively high birthrate (234.82 [95% CI 213.45–256.18] and 119.82 [95% CI 87.25–152.38] per 1000, respectively), especially compared to women who originated from America and Asia and the Pacific (51.99 [95% CI 33.39–70.60] and 64.88 [95% CI 58.68–71.08] per 1000, respectively) (see Table 2).

### 3.4. Teenage Pregnancies

During the study period, 72 teenage mothers gave birth while they lived in ASCs. Of these teenage mothers, 49 (68.1 percent) were unaccompanied minors. Compared to the Dutch population, the teenage birthrate among asylum-seeking women was 6.77 times higher (see Table 2) [22]. Most of these teenage mothers originated from Middle East/North Africa and East/Horn of Africa (28 and 21 respectively). The teenage birthrate was the highest among women from West/Central Africa and East/Horn of Africa (70.18 and 20.13 per 1000, respectively).

Compared to non-teenage mothers, teenage mothers were less often registered with a partner (45.8 vs 55.3 percent), and a short length of stay in The Netherlands at birth was relatively more common (66.7 vs 51.6 percent). Specifically, 66.7 percent of teenage mothers gave birth within 9 months of their stay in an ASC, compared to 51.6 percent of non-teenage mothers (see Table 3).

## 4. Discussion

This study presented an overview of childbirths in Dutch ASCs from 2016 to 2020, including maternal characteristics and the prevalence of previously described risk factors for adverse perinatal outcomes. We found that asylum seekers had a 2.15 times higher birthrate and a 6.77 times higher teenage birthrate compared to the Dutch population. Almost 70 percent of teenage mothers were unaccompanied minors, and 11.3 percent of all women were undocumented at the time of childbirth. Notably, more than half of all mothers and 66.7 percent of teenage mothers in this study were pregnant upon arrival in an ASC, with the highest number of total births in the second month after arrival. Only 55.1 percent of all mothers and 45.8 percent of teenage mothers were registered with a partner, and 69.5 percent of all women were relocated at least once during pregnancy. These findings offer important reflections into the origin of perinatal health inequities between asylum seekers, other migrants, and native populations.

The relatively high birthrate among asylum seekers in this study was likely related to limited access to and availability of sexual and reproductive health services throughout the process of forced migration [24,25,26,27]. In absolute numbers, most children in this study were born to mothers from the Middle East/North Africa, a region which includes common countries of origin among asylum seekers such as Syria and Iraq [28]. The highest birthrate was found among women from different African regions, which is in line with the UN estimate of 4.7 births per woman in sub-Saharan Africa, more than twice the level of any other world region [29,30].

Most women who gave birth during the study period were pregnant on arrival at an ASC. The peak in births in the second month after arrival indicated that most of these women were already in their third trimester at the date of registration. The arrival of pregnant women with a refugee background has been addressed by two recent Italian studies. In one study, 11 percent of all migrants arrived pregnant; in another study, 45 percent of pregnant women living in reception centers were pregnant on arrival [31,32]. In non-academic reports, humanitarian organizations raised concerns over the number and dire circumstances of pregnant women in refugee camps and documented a minimum of 27 deaths of pregnant migrants at European borders in the last decade [33,34,35]. To our knowledge, no research has studied the percentage of women that became pregnant before leaving their homelands or along the way. As women on the move are prone to gender-based violence, a substantial part of their pregnancies may be due to rape [10,11,32,36]. Regardless of how, when and where women became pregnant, antenatal care will mostly start late or get disrupted for women arriving pregnant in ASCs.

Overall, the number of childbirths decreased with increasing length of stay, which could be partially attributed to asylum seekers leaving ASCs. However, we found that a relatively high number of women became pregnant in the first 3 months after arrival in ASCs. Refugees’ hope that pregnancy may help to receive a residence permit may be one explanation for this relative peak in births between 9 and 12 months of stay [21]. Although the background and motives of having a baby shortly after reaching a destination country need to be further explored, these results stress the need for access to reproductive health services immediately after arrival.

A substantial part of the women who gave birth shortly after arrival most likely concerned undocumented women, who are legally entitled to shelter from 6 weeks prior to their due date to 6 weeks after birth in The Netherlands. As not all women use this option, for instance because they are unaware of the right to shelter or fear deportation, the 319 women in our study probably represent an underestimation of the number of undocumented women giving birth in The Netherlands. Compared to different European populations, poor perinatal health outcomes have been reported in undocumented migrants [37,38,39]. Although few studies have compared perinatal outcomes between documented and undocumented migrants, the intersection of a precarious legal status, jeopardized access to healthcare and systemic and social exclusion likely renders undocumented migrants a particularly vulnerable group of pregnant women in ASCs [3,38,39,40,41].

Considering the increased risks of sexual abuse and exploitation among young girls, the high percentage (66.7 percent) of teenage mothers in our study who arrived pregnant in ASCs was especially alarming [36,42]. Teenage pregnancy and childbirth have been linked to poor perinatal health outcomes and may have long-term negative socioeconomic consequences [43]. In line with earlier research, this study demonstrated a relatively high teenage birthrate among asylum seekers (17.80/1000) [21]. The high teenage birthrate in women from sub-Saharan Africa (SSA) corresponds to literature estimating that one in four adolescent girls in SSA gives birth before reaching 18 years old [43]. Young asylum seekers may be at increased risk of early and unintended pregnancies because of discontinued education, disrupted family structures or a lack of financial means and contraceptives [21,44].

Over half of the teenage mothers, and 44.9 percent of all mothers in this study were registered without a partner at the time of childbirth in an ASC. Moreover, 68.1 percent of all teenage mothers were unaccompanied minors. While social connectedness is not limited to (registered) civil status or having a guardian, these numbers suggested that social isolation may be significant among mothers in ASCs. Asylum seekers are often separated from family and friends, which adds to the complex reality of new parenthood simultaneously with resettlement in a new country. A lack of social support has consistently been shown to increase the risk of perinatal mental health disorders across general, but also refugee, populations [45,46]. For asylum seekers, single motherhood was identified as a specific risk factor for severe acute maternal morbidity [8]. A recent systematic review concluded that community building and a stimulating social network are key protective factors across interventions for refugee mothers [47].

Another finding in our study concerned the frequent relocations of pregnant women between ASCs. In our population, 69.5 percent of women were relocated at least once, and 28.2 percent were relocated two times or more during pregnancy. No conclusions can be drawn regarding the reasons for relocations, or how relocations may have affected the health or wellbeing of the pregnant women in our study. However, in a previous systematic review of qualitative evidence, the effects of relocations included discontinuity of care, repeated interventions and missed treatment leading to potentially dangerous medical situations [48]. Moreover, frequent or late relocations caused feelings of powerlessness, stress and anxiety among pregnant asylum seekers in multiple studies. Care providers reported how relocations frustrated the care process and interfered with the ability to form trusting relationships with their clients [12,48,49,50,51].

### 4.1. Strengths and Limitations

An important strength of this study regarded the unique source of data as provided by the COA. As such, we were able to consider all childbirths registered in ASCs, including multiple maternal, demographic, and social factors that appear relevant to perinatal health. To our knowledge, no previous studies have quantified relocations of asylum seekers during pregnancy. Since migrant perinatal health research has long failed to acknowledge the heterogeneity within migrant populations, our focus on residents of ASCs (including undocumented women and minors) represents another important strength of this study.

Several limitations should be considered in the interpretation of our results. Firstly, the available data only included maternal characteristics and no clinical outcomes of childbirths among residents of ASCs. Although a detailed population profile proves an important first step in recognizing risk factors and reproductive health needs, further research is needed to consider associations between maternal characteristics of asylum seekers and pregnancy outcomes. As abortive outcomes and stillbirths could not be included in this study, our study population represented an underestimation of the total population of pregnant women in ASCs.

No general health, lifestyle or obstetric care parameters could be included in this study besides maternal age, and only limited information related to the asylum process was available. Details on the length or status of the procedure, migration motives and language barriers could provide more insight into the situation of women who are pregnant while seeking asylum. The understudied subpopulation of undocumented migrants was part of our sample, but we could not disaggregate other characteristics of these women. Lastly, length of stay in ASCs may not represent the true duration of residence in The Netherlands for all women in this study, as only the latest date of registration in an ASC was available.

### 4.2. Policy and Research Recommendations

The high percentage of women pregnant on arrival in this study urges rapid referral pathways and support in navigating the maternity care system for women in ASCs. Healthcare professionals attending to asylum seekers should be aware that pregnancy may be unplanned and/or unwanted and be equipped to offer trauma-informed care. Education and empowerment with regards to sexual and reproductive health and rights should be facilitated for (teenage) asylum seekers and especially unattended minors. In addition, the relatively large percentage of (expectant) single mothers calls for programs and policy focused on social support. Given the psychosocial effects and discontinued care associated with relocations of pregnant asylum seekers, these should be kept to a minimum [50].

Future research should provide more insight into the prevalence of migration-related risk factors and their association with adverse pregnancy outcomes in refugee women. Studies should focus specifically on the effects of migration policies, housing, and integration of refugees on different maternal and perinatal health outcomes. Ultimately, to advance research and monitoring of otherwise invisible subpopulations, quality registration of migration indicators in care and the possibility to link these to pregnancy outcomes is key.

## 5. Conclusions

In conclusion, this study showed a high birthrate and a high prevalence of previously described risk factors associated with adverse pregnancy outcomes in the asylum-seeking population in The Netherlands. These risk factors include a high rate of teenage pregnancies, single motherhood, frequent relocations, and a short length of stay. We identified a substantial number of unaccompanied minors and undocumented women, who face additional barriers to perinatal care. The relationship between included characteristics and perinatal outcomes could not be determined in our study, since the latter were lacking from the data, and linkage to other datasets was not possible. This limitation stresses the importance of including migration-related indicators in perinatal health registration to support future interventions, policies, and research. Ultimately, our findings call for tailored and timely reproductive and perinatal healthcare for refugee women who simultaneously face the challenges of resettlement and pregnancy.

## Figures and Tables

**Table 1:** Childbirths and maternal characteristics among asylum seekers.

	*n* (%)
**Age**	
15–19	72 (2.5)
20–29	1540 (54.4)
30–39	1078 (38.1)
40–49	139 (4.9)
50+	2 (0.1)
**Regions of Origin**	
America	30 (1.1)
Asia and Pacific	417 (14.7)
Europe	361 (12.8)
Middle East/North Africa	957 (33.8)
East/Horn of Africa	528 (18.7)
West/Central Africa	458 (16.2)
Southern Africa	50 (1.8)
Unknown/stateless	30 (1.1)
**Registered with Partner**	
Yes	1560 (55.1)
No	1271 (44.9)
**Length of Stay**	
0–9 months	1471 (52.0)
9–12 months	409 (14.4)
12+ months	951 (33.6)
**Number of Relocations during Pregnancy**	
0	864 (30.5)
1	1169 (41.3)
2	439 (15.5)
3	235 (8.3)
4 or more	124 (4.4)
**Subgroups**	
Unaccompanied minors	49 (1.7)
Undocumented women	319 (11.3)

**Table 2:** Variation in (teenage) birthrates among asylum seekers between different regions of origin compared to the Dutch population.

	Women of Fertile Age (15–49)						Women Aged 15–19					
Region of Origin of the Mother	Women per 5 Years ^1,2^	Births in 5 Years	Birthrate per 1000 ^3^	95% CI Birthrate per 1000		Birth Ratio vs. NL	Women per 5 Years	Teenage Births in 5 Years	Teenage Birthrate per 1000 ^3^	95% CI Teenage Birthrate per 1000		Teenage Pregnancy Ratio vs. NL
				Lower	Upper					Lower	Upper	
**The Netherlands**	18,874,506	849,242	44.99	44.90	45.09	N/A	2,539,944	6678	2.63	2.57	2.69	N/A
**Asylum seekers**	30,194	2922	96.77	91.93	98.90	2.15	4045	72	17.80	13.69	21.91	6.77
America	577	30	51.99	33.39	70.60	1.16	62	0	0.00	0	0	0.00
Asia and Pacific	6489	421	64.88	58.68	71.08	1.44	699	7	10.01	2.60	17.43	3.81
Europe	3756	368	97.98	87.97	107.99	2.18	397	6	15.11	3.02	27.21	5.75
Middle East/North Africa	10,757	971	90.27	84.59	95.94	2.01	1479	28	18.93	11.92	25.94	7.20
East/Horn of Africa	5891	536	90.99	83.28	98.69	2.02	1043	21	20.13	11.52	28.75	7.66
West/Central Africa	1976	464	234.82	213.45	256.18	5.22	114	8	70.18	21.55	118.80	26.69
Southern Africa	434	52	119.82	87.25	152.38	2.66	42	0	0.00	0	0	0.00
Unknown/stateless	314	30	95.54	61.35	129.73	2.12	209	2	9.57	−3.69	22.83	3.64

^1^ The total population of asylum-seeking women of fertile age in the Dutch ASCs (2016–2020): sum of estimated person years 2016–2020. ^2^ The total population of women of fertile age in The Netherlands (2016–2020): sum of women per year 2016–2020 (source: CBS). ^3^ Birthrates for the Dutch population were calculated per 1000 women aged 15–49. For asylum seekers, birthrates were expressed per 1000 person years of women aged 15–49.

**Table 3:** Registration with partner and length of stay in teenage and non-teenage mothers.

	*n* (%)	Teenage Mothers	Non-Teenage Mothers *n* (%)
		*n* (%)	
**Asylum Seekers**	2831 (100)	72 (100)	2759 (100)
**Registered with Partner**			
Yes	1560 (55.1)	33 (45.8)	1527 (55.3)
Unknown	1271 (44.9)	39 (54.2)	1232 (44.7)
**Length of Stay in ASC at Childbirth**			
0–9 months	1471 (52.0)	48 (66.7)	1423 (51.6)
9–12 months	409 (14.4)	5 (6.9)	404 (14.6)
>12 months	951 (33.6)	19 (26.4)	932 (33.8)

## Data Availability

Restrictions apply to the availability of these data. Data were obtained from the Dutch Central Agency for the Reception of Asylum Seekers (in Dutch “Centraal Orgaan opvang Asielzoekers; COA”) and are available upon reasonable request from the authors with the permission of the COA.

## Appendix A

**Table A1:** Number of births and person years per year from 2016 to 2020.

Year	Number of Births	Person Years of Women Aged 15–49 in ASCs
2016	778	7708
2017	452	5412
2018	427	5052
2019	652	5824
2020	572	6198
Total	2881	30,194

## Appendix B

**Table A2:** Number of births per country of origin.

Regions of Origin	Countries	Number of Births
America *n* = 30	Brazil Colombia Cuba El Salvador Honduras Nicaragua Suriname Venezuela United States of America	1 4 5 3 2 1 3 10 1
Asia and Pacific *n* = 417	Kazakhstan Kyrgyzstan Uzbekistan China North Korea India Nepal Sri Lanka Bangladesh Indonesia Mongolia Myanmar Thailand Viet Nam Afghanistan Islamic Republic of Iran Pakistan	2 1 2 32 1 1 1 7 1 1 9 2 1 2 147 165 42
Europe *n* = 361	Armenia Azerbaijan Belarus Georgia Russian Federation Turkey Ukraine Austria Germany Italy Latvia Republic of Moldova (the) Romania Albania Bosnia and Herzegovina North Macedonia Serbia Kosovo Yugoslavia Sovjetunie	23 24 3 19 38 108 19 1 1 1 1 16 1 32 7 17 11 6 25 8
Middle East/North Africa *n* = 957	Iraq Israel State of Palestine Jordan Kuwait Lebanon Saudi Arabia Syrian Arab Republic United Arab Emirates Yemen Algeria Egypt Libya Mauritania Morocco Tunisia	182 4 1 9 4 13 7 620 6 29 4 24 26 1 23 4
East/Horn of Africa *n* = 528	Burundi Djibouti Eritrea Ethiopia Kenya Rwanda Somalia Sudan United Republic of Tanzania Uganda	11 1 236 111 6 3 70 22 5 63
West/Central Africa *n* = 458	Burkina Faso Cameroon Côte d’Ivoire Ghana Liberia Guinea Gambia Togo Benin Mali Niger Nigeria Senegal Sierra Leone	1 6 18 7 4 80 20 1 6 1 2 272 4 36
Southern Africa *n* = 50	Angola Democratic Republic of the Congo Madagascar Malawi Zimbabwe	13 32 2 1 2
Unknown/stateless *n* = 30	Unknown Stateless	28 2
